# A scoping review into the impact of animal imagery on pro-environmental outcomes

**DOI:** 10.1007/s13280-019-01271-1

**Published:** 2019-10-25

**Authors:** Laura Thomas-Walters, Claire McNulty, Diogo Veríssimo

**Affiliations:** 1 Durrell Institute of Conservation and Ecology, University of Kent, Giles Ln, Canterbury, CT2 7NZ UK; 2 National Geographic Society, 10 Hammersmith Grove, London, W6 7AP UK; 3 Oxford Martin Fellow, University of Oxford, Oxford, UK; 4 Department of Zoology, University of Oxford, Oxford, UK; 5 San Diego Zoo Institute for Conservation Research, Escondido, CA USA

**Keywords:** Animal image, Behaviour change, Conservation, Human behaviour, Marketing, Photo

## Abstract

With the recognition that most global environmental problems are a result of human actions, there is an increasing interest in approaches which have the potential to influence human behaviour. Images have a powerful role in shaping persuasive messages, yet research on the impacts of visual representations of nature is a neglected area in biodiversity conservation. We systematically screened existing studies on the use of animal imagery in conservation, identifying 37 articles. Although there is clear evidence that images of animals can have positive effects on people’s attitudes to animals, overall there is currently a dearth of accessible and comparable published data demonstrating the efficacy of animal imagery. Most existing studies are place and context-specific, limiting the generalisable conclusions that can be drawn. Transdisciplinary research is needed to develop a robust understanding of the contextual and cultural factors that affect how animal images can be used effectively for conservation purposes.

**Electronic supplementary material:**

The online version of this article (10.1007/s13280-019-01271-1) contains supplementary material, which is available to authorised users.

## Introduction

Most global environmental problems are a result of human actions (Amel et al. 2017; Green et al. 2019). From reducing demand for illegally traded wildlife products to promoting the use of renewable energy sources, tackling today’s major environmental threats comes down to influencing human behaviour. In recognition of this, the biodiversity conservation field has moved beyond the biological sciences and has incorporated the social sciences and humanities (Bennett et al. 2016; Teel et al. 2018). Researchers are now attempting to understand the cognitive, social, and motivational processes that inform behavioural models to provide insights into appropriate approaches for effective behaviour change (Schultz 2014; Reddy et al. 2016). This involves the use of a variety of theoretical and applied perspectives to quantify the non-material relationships between humans and wildlife (Echeverri et al. 2018).

Experiences of nature can have beneficial effects on a range of pro-conservation variables (as well as personal well-being), such as connectedness with nature and pro-environmental attitudes (Kellert 2002; Lumber et al. 2017). A sense of connection with nature through the formation of an affective and/or cognitive relationship is believed to create an appreciation and value for all life, transcending a utilitarian view of nature (Lumber et al. 2017). With increasing urbanisation, however, these direct experiences of nature are becoming less common, a disconnect that is particularly concerning considering the rapid urbanisation in biodiversity hotspots (Kellert 2002; Cohen 2006; Güneralp et al. 2015). Although there is increasing effort to make urban environments less harmful to wildlife, species are still being lost at an alarming rate and it is vital that we use every tool at our disposal to foster connections between people and wildlife in aid of conservation (Wachsmuth, Cohen and Angelo 2016; Dirzo et al. 2014).

There is increasing interest in approaches to change human behaviour, particularly the use of marketing techniques (Veríssimo 2019). Social marketing has been recognised as an applied conservation social science (Bennett et al. 2017) and the Society for Conservation Biology has now a working group dedicated to conservation marketing (Veríssimo 2019). Social marketing is not necessarily a panacea for conservation, but it can provide valuable guidance in designing effective behavioural interventions to be used in conjunction with other approaches that may be needed to catalyse individual, social, and political change (Corner and Randall 2011). Much of the research and discussion in this area so far has centred on the efficacy of different narratives and contextual effects. This has included work on framing of messages, messenger effects, and emotionalisation, integrating research from fields such as human wildlife conflict, science communication, and environmental education (Larson 2005; Draheim et al. 2011; Flemming et al. 2018; Veríssimo et al. 2018). However, despite the adage “a picture is worth a 1000 words”, there has been less investigation into the potential impacts of visual representations of wildlife. The superiority of pictures over text when it comes to information retention is long-established, as it is thought to engage deeper levels of semantic cognitive processing (Shepard 1967; Whitehouse et al. 2006; Hockley 2008). However, in spite of the substantial development of visual communication research in the past decade (see for example Huddy and Gunnthorsdottir 2000; Flemming et al. 2018), there is less research done on visual representations than on textual analysis (Göransson and Fagerholm 2018).

In many ways, society has become an experience economy organised around attention, and with the advent of colour printing and the internet, there is an abundance of visual imagery content (Schroeder 2006; Göransson and Fagerholm 2018). Images that are emotive and vivid have a powerful role to play in shaping persuasive messages (Joffe 2008). They can draw viewers in and aid in recall of important messages (Graber 1990), interacting with prior values and attitudes to shape affective and cognitive reactions (Domke et al. 2002). We respond to imagery directly, experiencing it in terms of emotions, mood, and intuitions (Branthwaite 2002). Images can be considered a convention-based symbolic system, a sophisticated form of visual rhetoric, with the power to transform our collective sensibilities (Scott 1994; Starrett 2003). In recognition of this, fields such as visual social semiotics use qualitative techniques and critical analysis to understand how images are deployed to convey certain meanings (Aiello 2006; Schroeder 2006). Sensory theories of visual communication, such as gestalt (perception of the whole rather than perceptions of individual parts) and constructivism (viewers construct images with quick eye movements combine to build a picture), attempt to explain how the brain processes visual cues such as colour or depth to help us understand why different images attract or distract us (Lester 2013).

Images are used by conservation organisations and the media to construct truths and communicate ideas (SeppÄNen and VÄLiverronen 2003; Hansen and Machin 2013; Göransson and Fagerholm 2018). Although the creation of symbolic representations of nature is ancient, the opportunities provided by technologies we have to reach people through the mass media are relatively new (Kellert 2002). Researchers have found that seeing pictures of nature may not be as effective as contact with actual nature, but they can have similar benefits and help the public to visualise abstract scientific concepts like biodiversity (SeppÄNen and VÄLiverronen 2003; Brooks et al. 2017). However, we need to think carefully about the types of images we use and the messages we are sending. Commonly used climate change symbols such as polar bears and melting ice caps, for instance, may be easily recognised, but frame climate change as a far-away issue, remote from everyday behaviour (Chapman et al. 2016). Rigorous evaluation is needed to empirically validate the methods that are used to change behaviour across different contexts, as there can often be unexpected results (Thomas-Walters and Raihani 2017).

This review will systematically screen existing studies on the use of animal imagery to foster conservation connections. Although many in situ conservation issues are best addressed through the management of habitats rather than single species, we have chosen to focus on images of animals specifically as they are most often used as conservation flagships both for behaviour change and for fundraising purposes (Simberloff 1998; Smith and Sutton 2008). Where there is sufficient data, we investigate the human emotional and cognitive response to images of animals, and how this varies across cultures, geographies, and demographic groups. *We* focus on where evidence is lacking and make recommendations for future research. This will help researchers and practitioners to assess the current scientific evidence when formulating conservation behaviour change interventions, and identify priority areas for further study.

## Methods

We searched two bibliographic databases Scopus and Web of Knowledge using the search strings given in Table 1. Searches were only undertaken in English and were not restricted by publication date. As these academic bibliographic databases do not contain grey literature (research produced by organisations outside of the traditional academic publishing channels), Google Scholar was also searched using the search strings given in Table 2. In addition, we sent a callout to approximately 250 members of the Society for Conservation Biology Conservation Marketing and Engagement, and Social Science Working Groups via email, and 2685 followers on Twitter.

**Table 1:** Final search strings for Scopus and Web of Knowledge

Population synonyms	Intervention synonyms	Effect synonyms	Field terms
Wildlife	Photograph*	Knowledge	Conservat*
Species	Picture*	Value*	Biodiversity
Animal*	Film	Attitude*	
		Behavio*	

Asterisks were used as wildcard operators, to broaden the search by returning all words with the same root stem. E.g., “photograph*” would return results containing “photograph”, “photographs”, and “photography”

**Table 2:** Google Scholar search strings

Animal	+	Photo	+	Behaviour OR behavior
Species		Image		Attitudes
		Picture		

Once the articles captured through the searches were compiled and duplicates removed, the titles and abstracts were screened and categorised according to the inclusion criteria (Table 3). Where there was doubt about whether or not an article met the inclusion criteria, it was retained for assessment during the full- text screening. Once documents had been screened on the basis of their titles and abstracts, all reasonable efforts were made to obtain full-text electronic or paper copies of the documents, including emailing corresponding authors. Articles which had passed the title and abstract screening but for which we were unable to obtain full-text copies were excluded, although this was only one study (Shuttleworth 1980). We then used the bibliographies of the articles returned from our database search to identify further relevant studies during the full-text screening.

**Table 3:** Screening inclusion criteria

Category 1—Responses to animal images	Category 2—Aesthetic preferences
Studies which test the effect of animal images on people’s conservation knowledge, values, attitudes, or behaviours	Studies which empirically test aesthetic preferences for and perceptions of species and landscapes using images

## Results

**Fig. 1:**
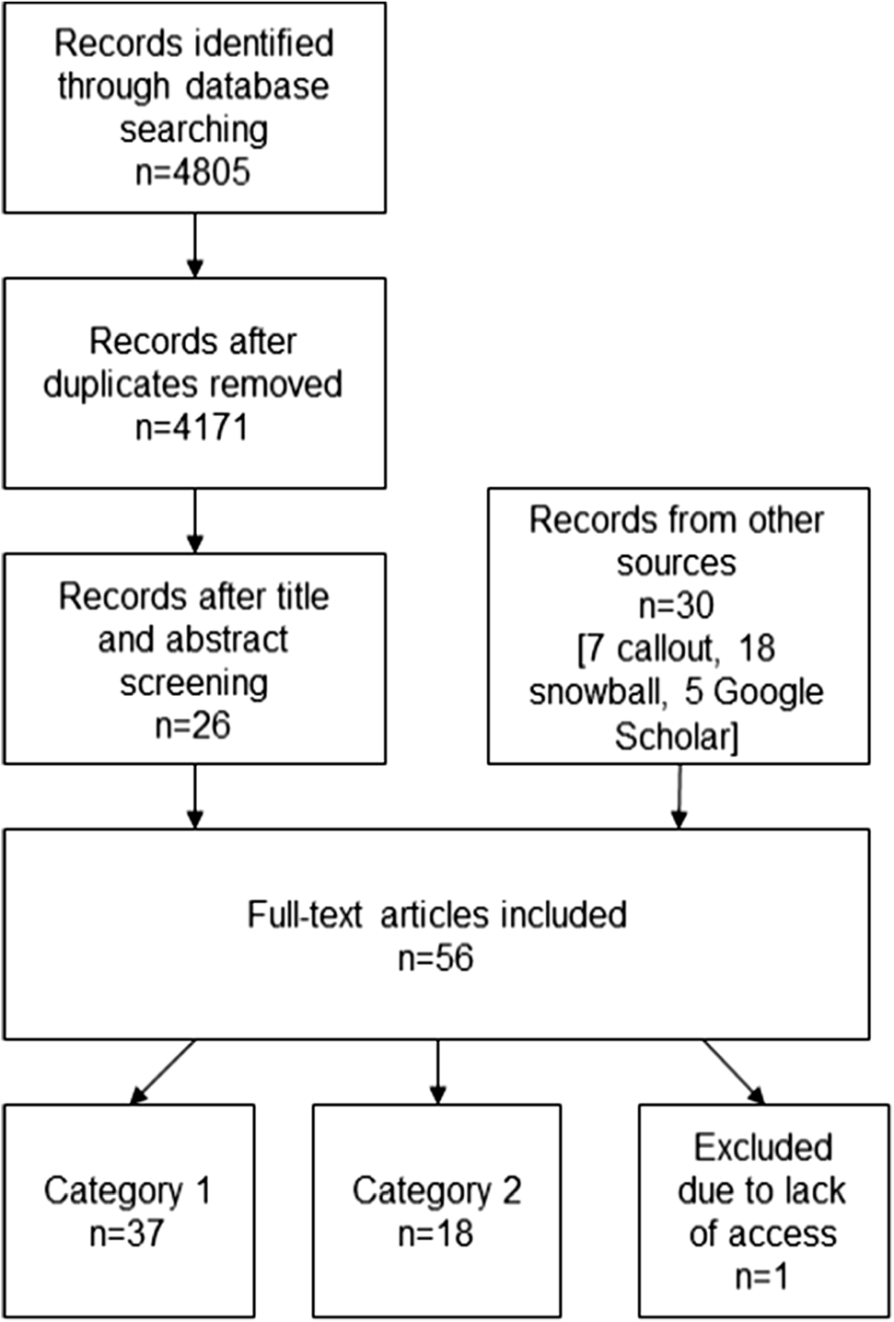
Flow diagram illustrating articles retrieved in initial search and articles included following subsequent screening and full-text assessment. Diagram stages adapted from PRISM guidance (Moher et al. 2009)

We identified 38 papers that empirically tested people’s responses to images of animals (Fig. 1). Full references for each paper can be found in Appendix S2.

**Fig. 2:**
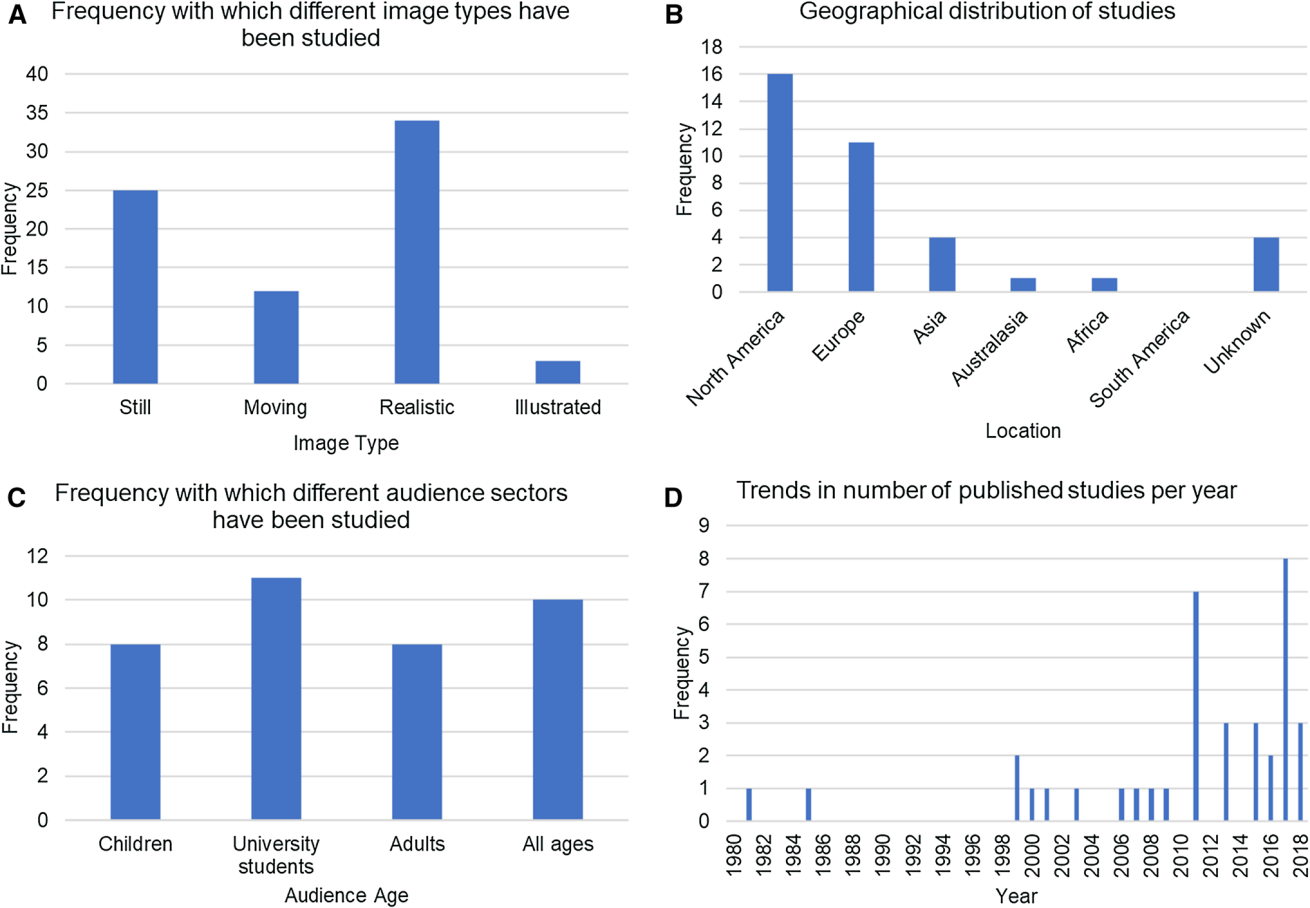
Variation in image type (**a**), geographic distribution (**b**), target audience (**c**), and year of publication (**d**) among published empirical studies identified through this review

The effects of a range of different visual media were examined, including documentaries, photography exhibitions, television commercials, media campaigns, and popular movies (Fig. 2). The majority focused on still (68%) images (photographs, drawings, etc.) rather than moving ones (documentaries, commercials, etc.), and realistic (92%) rather than illustrated. In terms of geographic representation, North America and Europe are the most studied cultures with 27 articles (73%), whereas Africa and South America had some of the fewest articles for their geographic extent. We found few articles prior to 1999 with a substantial increase after 2010. In addition, we identified a further 18 papers which looked at how preferences for visual attributes of species varied which we used to inform the later section on the relationship between human preferences and aesthetic appeal.

## Discussion

There is currently a dearth of accessible and comparable published data demonstrating the efficacy of animal imagery. We identified very few studies looking at the topic (Fig. 1) and most existing studies are place and context-specific, limiting the generalisable conclusions that can be drawn. Important variables that influence responses to visual conservation messages include culture, age, gender, education, and degree of urbanisation. Although some researchers have specifically examined the connections between visual triggers and conservation outcomes, others were interested in a more general sensitisation of participants to conservation. Still others only looked at the relationship between visual cues and animal-oriented behaviour, disassociated from conservation outcomes. Due to the highly disparate nature of the studies, it was not possible to organise the review by response variables, though we do identify any pro-conservation variables studied where applicable.

Some clear lessons do emerge, however. Images of animals can have positive effects on people’s attitudes to animals, altering their emotional responses and willingness to protect them (Kalof et al. 2011; Štefaniková and Prokop 2013; Kalof et al. 2016). Aesthetic appeal is a major contributor to these impacts. There are links between the amount of exposure to wildlife media and the way people behave and feel towards conservation, although the mechanism of this relationship is unclear. However, the literature is fairly disparate. The current research is not concentrated in areas where biodiversity is concentrated, and many types of images have been neglected, e.g. moving visual images such as videos.

### Comparing imagery styles and presentation

One aspect that has received relatively little attention in the literature is comparisons of different formats of engagement, such as different documentary styles or classroom lectures, with most studies focusing on the effects of photographs alone (see Fig. 2a). Both viewing a Cousteau Society documentary on marine mammals and listening to a science teacher’s presentation of the documentary’s script improved knowledge and attitudes about marine mammals for American adolescents (Fortner 1985). One study compared the effects of two different styles (traditional versus non-verbal) of nature documentaries featuring insects on Greek 12-year-olds (Barbas et al. 2009). Although both styles were equally effective in increasing empathetic feelings towards the environment, the non-verbal documentary was superior at developing environmental knowledge. No hypothesis was put forwards as to why the absence of a verbal or written narrative actually increased knowledge, and the neglect of any behavioural measures limits the usefulness of these investigations. It also raises questions regarding the effect of cultural context – would similar results have been found with Kenyan or American school children? There is no guarantee that these findings translate across cultures, limiting the general recommendations that can be made as a result of this research.

Other studies have tested the likeability and willingness to protect animals across different formats, such as cartoons and photographs. For example, adult Filipinos were significantly more willing to protect their national bird, the Philippine Eagle (*Pithecophaga jefferyi*) when shown coloured rather than black-and-white photographs (Labao et al. 2008). In another study, anthropomorphised cartoon illustrations rather than photographs may also increase interest and likeability (Louch et al. 1999; Osinski 2017).

When animal representations are placed in a visual context that is more typically associated with human representation, a form of portrait photography, perceptions of animals as individuals with a personality are enhanced (Kalof et al. 2011, 2016). This has resulted in both Canadian college students and visitors to a French museum experiencing an increase in feelings of kinship with animals after viewing (Kalof et al. 2011, 2016).

### The species effect - aesthetics, anthropomorphism, and charisma

Aesthetics is an important factor in how people engage with images of animals, and people are more willing to support an animal they find aesthetically pleasing (Gunnthorsdottir 2001; Knight 2008; Liordos et al. 2017). This is not particularly surprising as a bias in conservation towards flagship species, generally charismatic vertebrate, has long been acknowledged (Clucas et al. 2008; Smith and Sutton 2008; Ducarme et al. 2013). Online experiments show that conservation campaigns featuring appealing species (e.g. a polar bear) will receive higher donations compared to ones featuring unappealing species (e.g. a stonefly) (Thomas-Walters and Raihani 2017; Veríssimo et al. 2017). However, it is worth noting that contrary to this evidence, in an analysis of an offline large-scale fundraising campaign no effect of appeal or familiarity of species was found on monetary donations (Veríssimo et al. 2018). These differences in results may be due to differences in methods, limiting comparability.

There has been some research into what exactly are the traits that influence physical attractiveness in a species, such as a preference for brightly coloured animals and similarity to humans (i.e. anthropomorphism) (Barua et al. 2012; Prokop and Fančovičová 2013; Breuer et al. 2015). As an example of anthropomorphism, greater body mass and bigger and forward-facing eyes have been found to guide preferences (Tisdell and Wilson 2006; Martín-López et al. 2007; Knegterin et al. 2010; Veríssimo et al. 2018). Similarly, a human preference for baby schema may influence judgement of bird silhouettes also, with short necks and big eyes being the most appealing traits (Lišková and Frynta 2013). There is a limited evidence base on which to judge the role of anthropomorphism in influencing behaviour or even attitudes towards biodiversity, however, particularly in the case of biological groups such as plants that commonly receive less public attention (Root-Bernstein et al. 2013).

Although there is clear evidence that humans are influenced by aesthetic appeal, we should not necessarily focus campaigns on a single subset of animals. Too much focus on appealing, charismatic animals may lead to a neglect of other threatened species and contribute to conservation issues (Simberloff 1998; Fazey et al. 2005; Douglas and Winkel 2014). In addition, understanding factors that compensate for a lack of aesthetic appeal is important because many endangered species are not ideal flagships. For example, in one Swiss city, familiarity and ecological utility meant that the clover stem weevil (*Ischnopterapion virens*) outperformed the great spotted woodpecker as a flagship species for a hypothetical conservation project (Home et al. 2009). Other predictors of support include rarity and endangered status, suggesting that these are key features to be highlighted in any accompanying text (Gunnthorsdottir 2001; Angulo and Courchamp 2009; Schlegel and Rupf 2010). Including more information or increasing the marketing effort for an undesirable species can increase support, relative to other animals (Veríssimo et al. 2017; Curtin and Papworth 2018). Research on appeals featuring human subjects shows that people demonstrate a greater willingness to help identified individuals rather than unidentified, or statistical, victims (Jenni and Loewenstein 1997). However, this effect does not seem to translate to appeals featuring wildlife, where assigning individual animal names and identities does not increase donations (Thomas-Walters and Raihani 2017). One reason for this could be the authenticity of identified victims in charitable appeals—it may be easier to believe that an orphaned girl called Juanita genuinely exists and needs your help than Rosie the polar bear.

### Animal imagery in the media

There is a link between the amount of time spent watching wildlife programmes and the way people behave and feel towards the environment. For example, American adults and Hong Kong adolescents who watch more wildlife and environmental programmes perform more conservation behaviours and believe more in valuing nature for itself, rather than any utilitarian purpose (Holbert et al. 2003; Clark 2006; Lee 2011). This is not necessarily a causal relationship, however, and it is important to be aware that any potential causality could run both ways. Given that environmental concern is a strong positive predictor of nature show consumption (Holbert et al. 2003), it could simply be the case that those who are already willing to change their behaviour are more likely to be watching nature programmes. To investigate any causal relationships a robust impact evaluation study design would be required, as comparing the values and behaviours of those who watch wildlife media with those who do not is an invalid approach (Veríssimo et al. 2018). One alternative would be to explore behaviour in a lab game or on outcomes that are easily measured such as ‘nature connectedness’ or donations to conservation immediately following exposure (Arendt and Matthes 2016; Barbas et al. 2009; Zelenski et al. 2015).

Whether the portrayal of animals in popular culture has positive, negative, or neutral effects on people’s behaviour and attitudes towards conservation is debated, and the evidence is inconclusive and often lacking. Despite multiple claims in the media that Finding Nemo and the Harry Potter film franchise led to an increase in demand for pet clown fish and owls, impact evaluations find no evidence to support this narrative (Megias et al. 2017; Militz and Foale 2017). It has also been suggested that viral videos could also affect demand for wild animals, and one study analysed the YouTube comments on a video of a slow loris being tickled (Nekaris et al. 2013). The proportion of comments about wanting a pet loris decreased significantly over time, and more viewers expressed awareness about the inhumane removal of slow lorises’ teeth in the pet trade. The video itself was not educational, but the forum allowed for the spread of conservation and ecological facts. This is an example of the ad hoc nature of much of the available data, and the difficulty it poses for drawing conclusions. Whether a proportionate decrease in comments reflects an actual decrease in desire for a pet loris or just a change in social norms cannot be ascertained.

### Animals in anthropocentric settings

Showing animals in a context with humans generally has a negative effect on specific aspects of human–animal relationships. Americans feel greater continuity (viewed animals and humans as more similar) towards a companion animal that has been photographed alone (Carter 2011) and are less likely to believe that primates are threatened and are more likely to desire them as a pet if they are shown with a human nearby (Ross et al. 2011; Leighty et al. 2015). This may be because people are better able to connect with a companion animal when photographed alone by picturing themselves with it, while chimpanzees are perceived as less dangerous and more manageable when in contact with humans (Leighty et al. 2015). They are also more likely to desire chimpanzees as pets and less likely to donate to a conservation charity after watching a commercial featuring an “entertainment” chimpanzee, e.g. working in an office, rather than a chimpanzee conservation commercial or footage of wild chimpanzees (Schroepfer et al. 2011). However, a human setting is not always harmful—most American undergraduates expressed positive attitudes towards an image of a coyote lying on a human bed, potentially because they were reminded of a pet dog (Draheim et al. 2011). The framing of similarities between humans and animals affects our moral concern for others and comparing animals to humans can reduce speciesism (although comparing humans to animals may have negative effects; Costello and Hodson 2008; Bastian et al. 2012). It is important to note that variables such as human–animal continuity do not necessarily translate to pro-conservation behaviours, presumably the end-goal of most conservation campaigns, and that these studies were all conducted on American audiences. In countries where dogs do not hold such a central place in a family’s home, or where people have actually had contact with threatened species like chimpanzees, the effects may be very different.

### Emotive imagery

Studies in health psychology and climate change show that fear appeals need to be coupled with constructive information that enable people to respond, therefore avoiding the risk of overwhelming their target audience. Fear appeals are frequently used in social marketing, as they can help form behavioural intentions by causing people to perceive themselves as vulnerable (Das et al. 2003; de Hoog et al. 2005; Moser and Dilling 2007). However, they can also overwhelm viewers, resulting in a disengagement from the message through denial of the problem, and apocalyptic messaging can lead people to question whether the messenger is trustworthy (Witte and Allen 2000; Stoll-Kleemann et al. 2001; Moser and Dilling 2007). We found only one study in conservation which examined the use of images in a fear appeal, where American undergraduates were shown a video about whaling (Shelton and Rogers 1981). Behavioural intentions to help were higher when noxiousness (e.g. gory scenes of bodily injury to whales) and efficacy (e.g. scenes of a Greenpeace crew successfully saving whales) were increased.

Limited evidence exists that shocking imagery may be more effective at eliciting donations. For example, when asked to split donations between two photographs of rhinos, British adults gave more money to the image that was more upsetting and gory (Pestridge 2017). A picture of a dead and bloodied rhino was chosen over an alive but visibly injured one. However, the study failed to investigate contextual variables, such as culture, that could affect donation decisions. For instance, people who were more highly educated actually donated less to the more shocking image. This limits the broader usefulness of the findings. When humans are the subject of a charitable appeal, the display of negative emotions can affect the emotional intensity generated by images and result in significantly larger donations (Burt and Strongman 2005). Whether this extends to animal victims has yet to be studied.

Attitudes have both an affective and a cognitive component and addressing both components might be the most effective method of changing attitudes (Pearson et al. 2011). Empathy is a strong predictor of prosociality, and researchers have been exploring ways to increase empathy towards different victims (Schultz 2000). For example, when Spanish undergraduates viewing images of nature being harmed (either a bird or a tree) were instructed to take the perspective of the object being harmed rather than remain objective, their willingness to help nature increased (Berenguer 2007). Innate threat responses, however, e.g. in reaction to viewing images of snakes, spiders, or animals in a dangerous pose, interfere with the capacity to feel empathy and compassion (Davey 2011; Štefaniková and Prokop 2013; Bertels et al. 2017).

### Variation across demographics and cultures

Most studies focused on inhabitants of Europe and North America (see Fig. 2b), limiting comparisons to other nationalities. The only cross-cultural study found considerable overlap in assessment of python and boa beauty in photographs by adults from the Czech Republic and Papua New Guinea (Marešová et al. 2009). However, research from health communication shows that culture can affect the efficacy of a given intervention strategy and has an important role to play in audience segmentation, suggesting an urgent need for more investigation in this area (Kreuter and McClure 2004). Prior attitudes and values of the audience may also influence their receptivity to different messages (Domke et al. 2002). Emotive images of animals have the greatest effect on the most involved environmental supporters, and watching a nature documentary may only increase pro-environmental donation behaviour for viewers who already have a strong sense of connectedness (Huddy and Gunnthorsdottir 2000; Arendt and Matthes 2016). Few significant gender differences were found, with the exception of emotional reactions to different species in children (Schlegel and Rupf 2010; Borgi and Cirulli 2015; Schlegel et al. 2016). Young girls showed greater fear and disgust to images of certain animals, such as spiders, and this was associated with lower levels of affinity. Although a range of ages have been examined (see Fig. 2c), none have attempted to determine whether images of animals affect, for example children differently than adults.

## Key recommendations for future research

Studies on the effects of animal images are few but increasing (*r*_*t*_*=* .455, *p* = .0003), as shown in Fig. 2d, and their designs and objectives are disparate and difficult to compare. Moreover, they have almost exclusively been conducted on a fairly narrow subset of Western audiences (see Fig. 2). Research in behavioural science shows that there is substantial variability in experimental results across populations, and this lack of diversity in research participants is concerning as both culture and education level may be important factors in determining responses to images of animals (Henrich et al. 2010). Attitudes are context-contingent, and there can be large differences between Western and non-Western cultural contexts (Riemer et al. 2014). Exploring how responses to narratives and visuals differs across cultures should be a top priority, which could require a deeper understanding of varying lay theories that people hold about nature. There is scope for transdisciplinary research incorporating fields such as neuroscience, psychology, and social marketing to develop a consolidated understanding of the different contextual and cultural factors that affect how animal images can be used effectively and cross-culturally in social marketing for conservation purposes, including when visual communication is less applicable. An investigation into the rationales used by non-governmental environmental agencies in the design of their campaign materials could also be illuminating.

Finally, we need to move beyond solely assessing attitudes or social media engagement to investigating actual or intended behaviour change. For instance, animal images may increase social media interest in a news story, but it is often unclear whether indicators such as likes, retweets, or even online pledges, actually translate to real-world behaviour changes (Curtin and Papworth 2018; Wu et al. 2018). Changes in knowledge alone are rarely sufficient to affect behaviour, and there is frequently a sizeable gap between intentions and actual behaviour (Kollmuss and Agyeman 2002; Sheeran 2002; Webb and Sheeran 2006). Integrating behavioural theory into campaigns, including drivers such as interpersonal communication, is necessary to achieve behaviour change (Green et al. 2019). Many papers failed to establish causal attribution, instead uncovering correlations between exposure to images of animals and a change in knowledge, attitudes, or behaviour. Very few also attempted to explore the psychological mechanisms behind the variable of interest, to elucidate not just whether a certain image had an effect but also why. The impact of specific image attributes was a relatively neglected area, as is the combination of narratives with images. Improving experimental designs may help us to elicit why an intervention succeeds or fails, and identify the conditions under which any causal effect arises (Baylis et al. 2016). This is an area in which fields such as international development have been leading the way, for example with the use of credible counterfactuals and theory-based evaluation. Conservation science should follow in their footsteps when it comes to adopting best practices in impact evaluation (Banerjee and Duflo 2009; White 2009; Baylis et al. 2016). If the integration of visual media into our daily lives continues to increase, then understanding its use as a tool to communicate the importance of wildlife will become ever more crucial.

## Electronic supplementary material

Below is the link to the electronic supplementary material.

Supplementary material 1 (PDF 111 kb)
