# Supplementary material for: A scoping review into the impact of animal imagery on pro-environmental outcomes
Source: Ambio. 2019 Oct 25;49(6):1135–45. doi: 10.1007/s13280-019-01271-1 (PMC7127996; doi:10.1007/s13280-019-01271-1)
Supplement: Supplementary file 1 — Supplementary material 1 (PDF 111 kb) [file 13280_2019_1271_MOESM1_ESM.pdf]

Ambio

Electronic Supplementary Material

This supplementary material has not been peer reviewed

Title: **A scoping review into the impact of animal imagery on pro-environmental outcomes**

Laura Thomas-Walters, Claire McNulty, & Diogo Veríssimo

Appendix S1: Full methodology

Searches

Search terms were tested for the extent to which they provided related and meaningful results in SciVerse's Scopus (www.scopus.com), Thomson Reuter's Web of Knowledge (www.webofknowledge.com) and Google Scholar (scholar.google.co.uk). The search strings are given in Table 1. Search strings included a combination of population synonyms (those relating to animals), intervention synonyms (those relating to images), effect synonyms (those relating to measurable changes), and discipline terms (those relating to conservation).

The following bibliographic databases were searched on 18/05/18 for publications: Scopus and Web of Knowledge. Both databases cover natural and social sciences, and Scopus is the largest abstract and citation database of peer-reviewed literature. Searches were only undertaken in English and were not restricted by publication date. The search strings were used to search titles, abstracts and keywords in Scopus, and to search the field code "Topic" which includes title, abstract and keywords in Web of Knowledge. The reference manager Mendeley was used to manage the full text documents (Elsevier 2016).

Bibliographic databases may not always have research published in the last 6 months and do not contain grey literature (research produced by organisations outside of the traditional academic publishing channels), so Google Scholar was also searched. Due to limitations of advanced searching on this platform, simple search strings such as 'animal photo behaviour OR behavior'' had to be used. 'AND' operators were not necessary as Google Scholar applies them by default. The first 100 search results for each search were reviewed online. Only published reports and studies are indexed in the selected online databases, so any "inhouse" reports commissioned by non-governmental environmental were likely inaccessible. In addition, we sent

a callout to approximately 250 members of the Society for Conservation Biology Conservation Marketing and Engagement, and Social Science Working Groups via email, and 2685 followers on Twitter.

Article screening

Once the articles captured through the searches were compiled and duplicates removed, the titles and abstracts were screened and categorised according to the inclusion criteria (Table 3). Where there was doubt about whether or not an article met the inclusion criteria, it was retained for assessment during the full text screening. Screening was carried out using Abstrackr (http://abstrackr.cebm.brown.edu), an online screening program for systematic reviewers (Wallace *et al*., 2012).

Once documents had been screened on the basis of their titles and abstracts, all reasonable efforts were made to obtain full text electronic or paper copies of the documents, including emailing corresponding authors. Articles which had passed the title and abstract screening but for which we were unable to obtain full text copies were excluded, although this was only one study from 1980 (Shuttleworth, 1980). We then used snowball sampling to identify further relevant studies in the bibliographies of the articles returned from our database search during the full-text screening.

Appendix S2: Results of the literature search

<u>Category 1 – Empirical studies</u>

Arendt, F., & Matthes, J. (2016). Nature documentaries, connectedness to nature, and pro-environmental behavior. *Environmental Communication*, *10*(4), 453–472. http://doi.org/10.1080/17524032.2014.993415

Barbas, T. A., Paraskevopoulos, S., & Stamou, A. G. (2009). The effect of nature documentaries on students' environmental sensitivity: A case study. *Learning, Media and Technology*, *34*(1), 61–69. http://doi.org/10.1080/17439880902759943

Berenguer, J. (2007). The effect of empathy in pro-environmental attitudes and behaviors. *Environment and Behavior*, *39*(2), 269–283.

Carter, A. (2011). *Attitude change regarding animal abuse in adults the effect of education and visual aids*. University of Central Florida.

Clark, F. J. (2006). *Effects of watching wildlife television on wildlife conservation behavior*. University of Washington.

Curtin, P., & Papworth, S. (2018). Increased information and marketing to specific individuals could shift conservation support to less popular species. *Marine Policy*, *88*(November 2017), 101–107. http://doi.org/10.1016/j.marpol.2017.11.006

Draheim, M. M., Rockwood, L. L., Guagnano, G., & Parsons, E. C. M. (2011). The impact of information on students' beliefs and attitudes toward coyotes. *Human Dimensions of Wildlife*, *16*(1), 67–72. http://doi.org/10.1080/10871209.2011.536911

Eagles, P. F. J., & Demare, R. (1999). Factors influencing children’s environmental attitudes. *Journal of Environmental Education*, *30*(4), 33–37. http://doi.org/10.1080/00958969909601882

Flemming, D., Cress, U., Kimmig, S., Brandt, M., & Kimmerle, J. (2018). Emotionalization in science communication: The impact of narratives and visual representations on knowledge gain and risk perception. *Frontiers in Communication*, *3*(3), 1–9. http://doi.org/10.3389/fcomm.2018.00003

Fortner, R. W. (1985). Relative effectiveness of classroom and documentary film presentations on marine mammals. *Journal of Research in Science Teaching*, *21*(2), 115–126.

Gunnthorsdottir, A. (2001). Physical attractiveness of an animal species as a decision factor for its preservation. *Anthrozoos*, *14*(4), 204–214. http://doi.org/10.2752/089279301786999355

Holbert, R. L., Kwak, N., & Shah, D. V. (2003). Environmental concern, patterns of television viewing, and pro-environmental behaviors: Integrating models of media consumption and effects. *Journal of Broadcasting & Electronic Media*, *47*(2), 177–196. http://doi.org/10.1207/s15506878jobem4702

Huddy, L., & Gunnthorsdottir, A. H. (2000). The persuasive effects of emotive visual imagery: Superficial manipulation or the product of passionate reason. *Political Psychology*, *21*(4), 745–778. http://doi.org/10.1111/0162-895X.00215

Kalof, L., Zammit-Lucia, J., Bell, J., & Granter, G. (2016). Fostering kinship with animals: animal portraiture in humane education. *Environmental Education Research*, *22*(2), 203–228. http://doi.org/10.1080/13504622.2014.999226

Kalof, L., Zammit-Lucia, J., & Kelly, J. R. (2011). The meaning of animal portraiture in a museum setting: Implications for conservation. *Organization and Environment*, *24*, 150–174. http://doi.org/10.1177/1086026611412081

Labao, R., Francisco, H., Harder, D., & Santos, F. I. (2008). Do colored photographs affect willingness to pay responses for endangered species conservation? *Environmental and Resource Economics*, *40*, 251–264. http://doi.org/10.1007/s10640-007-9151-2

Lee, K. (2011). The role of media exposure, social exposure and biospheric value orientation in the environmental attitude-intention-behavior model in adolescents. *Journal of Environmental Psychology*, *31*(4), 301–308. http://doi.org/10.1016/j.jenvp.2011.08.004

Leeds, A., Lukas, K. E., Kendall, C. J., Slavin, M. A., Ross, E. A., Robbins, M. M., … Bergl, R. A. (2017). Evaluating the effect of a year-long film focused environmental education program on Ugandan student knowledge of and attitudes toward great apes. *American Journal of Primatology*, *79*(8), 1–9. http://doi.org/10.1002/ajp.22673

Leighty, K. A., Valuska, A. J., Grand, A. P., Bettinger, T. L., Mellen, J. D., Ross, S. R., … Ogden, J. J. (2015). Impact of visual context on public perceptions of non-human primate performers. *PLoS ONE*, *10*(2), 1–6. http://doi.org/10.1371/journal.pone.0118487

Liordos, V., Kontsiotis, V. J., Anastasiadou, M., & Karavasias, E. (2017). Effects of attitudes and demography on public support for endangered species conservation. *Science of the Total Environment*, *595*, 25–34. http://doi.org/10.1016/j.scitotenv.2017.03.241

Louch, J., Price, E. C., Esson, M., & Feistner, A. T. C. (1999). The effects of sign styles on visitor behaviour at the orang-utan enclosure at Jersey zoo. *Dodo: Journal of the*

Wildlife Preservation Trusts, *35*(January 1999), 134–150. Retrieved from http://search.ebscohost.com/login.aspx?direct=true&db=psyh&AN=2001-00027-003&site=ehost-live&scope=site

Megias, D. A., Anderson, S. C., Smith, R. J., & Veríssimo, D. (2017). Investigating the impact of media on demand for wildlife: A case study of Harry Potter and the UK trade in owls. *PLoS ONE*, *12*(10), 1–13. http://doi.org/10.1371/journal.pone.0182368

Militz, T. A., & Foale, S. (2017). The “Nemo Effect”: Perception and reality of Finding Nemo’s impact on marine aquarium fisheries. *Fish and Fisheries*, 1–11. http://doi.org/10.1111/faf.12202

Nekaris, B. K. A. I., Campbell, N., Coggins, T. G., Rode, E. J., & Nijman, V. (2013). Tickled to death: Analysing public perceptions of “cute” videos of threatened species (slow lorises - Nycticebus spp.) on Web 2.0 Sites. *PLoS ONE*, *8*(7), e69215. http://doi.org/10.1371/journal.pone.0069215

Nijman, V., & Nekaris, K. A. I. (2017). The Harry Potter effect: The rise in trade of owls as pets in Java and Bali, Indonesia. *Global Ecology and Conservation*, *11*, 84–94. http://doi.org/10.1016/j.gecco.2017.04.004

Osinski, B. (2017). *What’s the Draw: Illustrating the Impacts of Cartoons Versus Pohotographs on Attitudes and Behavioral Intentions for Wildlife Conservation*. PhD Thesis. Purdue University.

Papworth, S. K., Nghiem, T. P. L., Chimalakonda, D., Posa, M. R. C., Wijedasa, L. S., Bickford, D., & Carrasco, L. R. (2015). Quantifying the role of online news in linking conservation research to Facebook and Twitter. *Conservation Biology*, *29*(3), 825–833. http://doi.org/10.1111/cobi.12455

Pearson, E., Dorrian, J., & Litchfield, C. (2011). Harnessing visual media in environmental education: Increasing knowledge of orangutan conservation issues and facilitating sustainable behaviour through video presentations. *Environmental Education Research*, *17*(6), 751–767. http://doi.org/10.1080/13504622.2011.624586

Pestridge, E. (2017). *The role of shock imagery in non-governmental organisations and media campaigns surrounding the rhino poaching crisis*. University of Kent.

Prokop, P., & Fančovičová, J. (2013). Does colour matter? The influence of animal warning coloration on human emotions and willingness to protect them. *Animal Conservation*, *16*(4), 458–466. http://doi.org/10.1111/acv.12014

Ross, S. R., Vreeman, V. M., & Lonsdorf, E. V. (2011). Specific image characteristics influence attitudes about chimpanzee conservation and use as pets. *PLoS ONE*, *6*(7), 1–5. http://doi.org/10.1371/journal.pone.0022050

Schroepfer, K. K., Rosati, A. G., Chartrand, T., & Hare, B. (2011). Use of “entertainment” chimpanzees in commercials distorts public perception regarding their conservation status. *PLoS ONE*, *6*(10), 1–8. http://doi.org/10.1371/journal.pone.0026048

Shelton, M. Lou, & Rogers, R. W. (1981). Fear□arousing and empathy□arousing appeals to help: The pathos of persuasion. *Journal of Applied Social Psychology*, *11*(4), 366–378. http://doi.org/10.1111/j.1559-1816.1981.tb00829.x

Stefanikova, S., & Prokop, P. (2013). Introduction of the concept of adaptive memory to science education: Does survival threat influence our knowledge about animals? *Journal of Environmental Protection and Ecology*, *14*(3 A), 1403–1414.

Štefaniková, S., & Prokop, P. (2015). Do we believe pictures more or spoken words? How specific information affects how students learn about animals. *Eurasia Journal of*

Mathematics, Science and Technology Education, *11*(4), 725–733. http://doi.org/10.12973/eurasia.2015.1380a

Thomas-Walters, L., & Raihani, N. J. (2017). Supporting conservation: The roles of flagship species and identifiable victims. *Conservation Letters*, *10*(5), 581–587. http://doi.org/10.1111/conl.12319

Wu, Y., Xie, L., Huang, S. L., Li, P., Yuan, Z., & Liu, W. (2018). Using social media to strengthen public awareness of wildlife conservation. *Ocean and Coastal Management*, *153* (2017), 76–83. http://doi.org/10.1016/j.ocecoaman.2017.12.010

Category 2 – Aesthetic preferences

Angulo, E., & Courchamp, F. (2009). Rare species are valued big time. *PLoS ONE*, *4*(4), e5215. http://doi.org/10.1371/journal.pone.0005215

Batt, S. (2009). Human attitudes towards animals in relation to species similarity to humans: A multivariate approach. *Bioscience Horizons*, *2*(2), 180–190. http://doi.org/10.1093/biohorizons/hzp021

Borgi, M., & Cirulli, F. (2015). Attitudes toward animals among kindergarten children: Species preferences. *Anthrozoos*, *28*(1), 45–59. http://doi.org/10.2752/089279315X14129350721939

Breuer, G. B., Schlegel, J., & Rupf, R. (2015). Selecting insects as flagship species for Beverin Nature Park in Switzerland - a survey of local school children on their attitudes towards butterflies and other insects. *Eco.Mont*, *7*(1), 5–16. http://doi.org/10.1553/eco.mont-7-1s5

Home, R., Keller, C., Nagel, P., Bauer, N., & Hunziker, M. (2009). Selection criteria for flagship species by conservation organizations. *Environmental Conservation*, *36*(02), 139. http://doi.org/10.1017/S0376892909990051

Knegterin, E., Van Der Windt, H. J., & Schoot Uiterkamp, A. J. M. (2010). Public decisions on animal species: Does body size matter? *Environmental Conservation*, *38*(1), 28–36. http://doi.org/10.1017/S0376892910000755

Knight, A. J. (2008). “Bats, snakes and spiders, Oh my!” How aesthetic and negativistic attitudes, and other concepts predict support for species protection. *Journal of Environmental Psychology*, *28*(1), 94–103. http://doi.org/10.1016/j.jenvp.2007.10.001

Lišková, S., & Frynta, D. (2013). What determines bird beauty in human eyes? *Anthrozoos*, *26*(1), 27–41. http://doi.org/10.2752/175303713X13534238631399

Excluded due to lack of access:

Shuttleworth, S. (1980). The use of photographs as an environmental presentation medium in landscape studies. Journal of Environmental Management, 11, 61-76.
